# Guidelines for the practice and performance of manipulation under anesthesia

**DOI:** 10.1186/2045-709X-22-7

**Published:** 2014-02-03

**Authors:** Robert Gordon, Edward Cremata, Cheryl Hawk

**Affiliations:** 1 Cornerstone Professional Education, Inc, 4002 Streamlet Way, 28110 Monroe, NC, USA; 2 Palmer College of Chiropractic West, San Jose, CA, USA; 3 Fremont Chiropractic Group, Fremont, CA, USA; 4 Logan University, 1851 Schoettler Rd 63017 Chesterfield, MO, USA

**Keywords:** Manipulation under anesthesia, Chiropractic, Spinal manipulation, Spine-related pain

## Abstract

**Background:**

There are currently no widely accepted guidelines on standards for the practice of chiropractic or manual therapy manipulation under anesthesia, and the evidence base for this practice is composed primarily of lower-level evidence. The purpose of this project was to develop evidence-informed and consensus-based guidelines on spinal manipulation under anesthesia to address the gaps in the literature with respect to patient selection and treatment protocols.

**Methods:**

An expert consensus process was conducted from August-October 2013 using the Delphi method. Panelists were first provided with background literature, consisting of three review articles on manipulation under anesthesia. The Delphi rounds were conducted using the widely-used and well-established RAND-UCLA consensus process methodology to rate seed statements for their appropriateness. Consensus was determined to be reached if 80% of the 15 panelists rated a statement as appropriate. Consensus was reached on all 43 statements in two Delphi rounds.

**Results:**

The Delphi process was conducted from August-October 2013. Consensus was reached on recommendations related to all aspects of manipulation under anesthesia, including patient selection; diagnosis and establishing medical necessity; treatment and follow-up procedures; evaluation of response to treatment; safety practices; appropriate compensation considerations; and facilities, anesthesia and nursing standards.

**Conclusions:**

A high level of agreement was achieved in developing evidence-informed recommendations about the practice of chiropractic/manual therapy manipulation under anesthesia.

## Introduction

Spinal manipulation under anesthesia (MUA) is a procedure that was originally practiced by orthopedic surgeons and osteopathic physicians for the treatment of spinal pain since the late 1930’s [1,2]. Since the 1960’s, Doctors of Chiropractic (DC) have come to perform the majority of spinal MUA procedures [3]. Fibrosis Release Procedures is a term which includes MUA and perhaps better describes the comprehensive nature of the procedures used by DCs in performing MUA, since more than spinal manipulation is involved [4].

There are currently no widely accepted guidelines on standards for chiropractic MUA. The 1993 Guidelines for Chiropractic Quality Assurance and Practice Parameters considered MUA “equivocal”, and these guidelines have not been updated since 1993 [5]. In 2012, the American Association of Manipulation Under Anesthesia Providers (AAMUAP), a multidisciplinary panel of MUA experts, developed a set of guidelines for the practice, and educational parameters for MUA. Members of the organization undertook a further effort to develop a set of evidence-informed and consensus based guidelines developed by a panel of multidisciplinary experts, including MUA practitioners as well as experts who were not MUA practitioners. The results of this consensus process are presented in this article.

Although MUA has been said by some authorities to be “a reasonable method of treating certain patients with spinal pain”, [2] evidence for its effectiveness is limited, with few controlled studies. However, the studies that exist, the majority of which are case series, have shown positive results [2,3,6]. In the absence of higher levels of evidence, or when the published literature does not provide adequate detail about management parameters, formal consensus by experts can be useful [7,8].

### Indications for MUA

A concern in providing MUA is the lack of standardized protocols for patient selection [6]. Selecting the patient who will benefit most from MUA is essential to the success of the procedure, yet selection criteria have not been investigated thoroughly [6]. Generally, spinal MUA is used for patients who suffer from chronic nonspecific mechanical spine-related pain who have been minimally responsive (not reaching the expected level of outcome) to previous conservative therapy; this is considered a treatment failure for conservative therapy [3,9,10]. Etiology of their pain may be disc bulge/herniation, chronic recurrent sprain/strain, failed back surgery, or myofascial pain syndromes. The procedure is considered by many practitioners to be beneficial for the patient who has muscle spasm accompanied with pain and loss of terminal joint range-of-motion. These types of patients typically respond well to manipulation/physical therapy/exercise, but their relief may only be temporary.

Hallmarks for choosing a patient for MUA are 1) the presence of intersegmental and/or global recalcitrant motion restrictions that are thought to be fibrosis maintained, and 2) the unsuccessful attempt at more conservative measures that have included in-office spinal manipulation [4].

### Description of MUA procedures and follow up care

Another concern is the lack of standardization of MUA procedures and follow-up care [6]. It is well-established that MUA requires an interdisciplinary team which includes an anesthesiologist, an operating room (OR) nurse and a DC or other qualified manual therapy physician [4]. It is also generally accepted that the phases of MUA are 1) sedation; 2) manipulative procedures; 3) additional stretching/traction procedures; 4) follow-up in-office care without sedation [4].

#### Sedation

Monitored Anesthesia Care (MAC) is used, most frequently Diprivan (Propofol) and Versed [11].

#### Manipulative and additional procedures

The patient is taken through passive spinal, hip, shoulder, and extra spinal extremity ranges of motion, determined by the treating physician. Specific spinal manipulation is performed when the elastic barrier of resistance and segmental end range of motion is achieved. Stretching of the paraspinal and surrounding supportive musculature is performed to promote cervical, thoracic, lumbar, lumbopelvic and extra spinal flexibility in conjunction with attempting to restore proper kinetic motion. The patient is then awakened from the anesthesia which usually occurs minutes after the Diprivan (propofol) is stopped. They are then taken to recovery and monitored until full recovery has occurred. The patient is then discharged to rest until post MUA therapy is begun later the same day (or in as short a time as possible following MUA).

#### Follow-up care without sedation

Post MUA therapy is an essential part of the MUA procedure and is accomplished the same day, if possible. Post MUA therapy consists of warming up the involved areas, passive stretching as was accomplished in the MUA procedure, followed by interferential stimulation and cryotherapy. The patient is then sent home to rest. This procedure is repeated serially in most cases by having the patient return to the facility the next day and the following day(s). The average number of days for the MUA procedure to accomplish the desired outcome has been shown to be between 2-4 days [12,13]. The concept is that increasing movement each day in incremental amounts accomplishes the desired increase in range of movement and decreases pain far better than spending large amounts of time in one day to achieve the same result. This protocol for post MUA therapy is repeated 7-10 days after the final MUA followed by pre-rehabilitation and then formal rehabilitation for 3-6 weeks. Additional reduction in soreness and mild edema with an increase in range of motion has been noted when small, portable, multi-modality interferential/NMES/HVPC or TENS devices are applied in the OR immediately following the MUA and when the patients are sent home with these units as part of the post MUA therapy [12,13]. The rehabilitation program continues for 3-6 weeks following the MUA procedure to give the patient time to recover to pre-injury status. Marked improvement (80-97%) has been the general rule when the properly selected cases have received this procedure [14,15].

### Evidence for MUA treatment effects

A PubMed literature search using the term “manipulation under anesthesia” found 2 systematic reviews (2002 [2] and 2008 [3]) and one narrative review, [6] and no articles that were not addressed in the reviews [2,3,6]. The secondary sources (reviews) [2,3,6] were the primary references used for evaluating the evidence related to MUA, with emphasis on the most recent review (2013) [6]. Although it did not claim to be a systematic review, it did evaluate the strength of the existing evidence on the topic [6]. The evidence was assessed using the scheme described in the 2003 *Journal of Bone & Joint Surgery,*[16] which is commonly used in musculoskeletal medicine [17]. Definitions of the levels of evidence in this scheme are summarized in Table 1.

**Table 1:** Definition of levels of evidence for treatment results*

**Level I**	**Level II**	**Level III**	**Level IV**	**Level V**
high-quality RCT	Prospective cohort	CC	Case series	Expert opinion
SR of high-quality RCTs	Poor-quality RCT	Retrospective cohort		Case reports^1^
	SR of above study types	SR of above study types		

*Source: Wright JG, Swiontkowski MF, Heckman JD. Introducing levels of evidence to the journal. *J Bone Joint Surg Am.* Jan 2003;85-A(1):1-3.

*Abbreviations*: *RCT* randomized controlled trial, *SR* systematic review, *CC* case control study.

^1^For this project, case reports were classified as the same level as expert opinion.

The evidence for treatment effects of MUA consisted of Levels II, IV and V. Level II evidence included three prospective cohort studies and [18-20] three reviews (narrative review, 2013) [6] and (systematic reviews 2008 [3] and 2002) [2]. The remaining published literature on MUA consisted of Level IV studies (case series) and Level V studies (case reports and expert opinion) [6]. Overall, positive effects were noted for MUA in appropriately selected patients; however, the absence of control groups make it impossible to make a definitive assessment [2].

#### Evidence related to safety of MUA

No serious adverse effects were noted in any of the published studies of MUA treatment by chiropractors [3,4].

The purpose of this project was to develop evidence-informed and consensus-based guidelines on spinal MUA to address the gaps in the literature with respect to patient selection and treatment protocols in particular.

## Methods

### Preparation for Delphi panel

All three published reviews [2,3,6] were provided to the Delphi panel at the beginning of the project as background documents. The core committee, two of whom are experienced MUA practitioners who have been active in guideline development for MUA and one who is experienced in conducting consensus projects for guideline development, developed 43 seed statements, based on previous MUA guidelines and the background documents.

### Delphi consensus panel

The project was determined to be exempt (P/N 2013-017) by the Institutional Review Board of Life Chiropractic College West prior to conducting the Delphi process. An expert consensus process was conducted using the Delphi method. Because a Delphi panel is made up of experts, we selected individuals on the basis of their established expertise in the area of spine-related care. We identified both individuals who practice MUA and those who provide spinal care without MUA, to avoid bias toward MUA practice. We also included laypersons familiar with spine-related care, such as insurance specialists and attorneys. A list of 24 panelists to be invited included healthcare providers who had published on MUA, were MUA practitioners, were experienced DCs who did not practice MUA but had a practice emphasis in chronic spinal pain and were familiar with guideline development, and several laypersons with healthcare experience such as insurance specialists and attorneys. Medical doctors (MD) (anesthesiologists and other specialists), osteopathic and chiropractic physicians were included, as well as registered nurses (RNs). A total of 16 panelists accepted, of which 10 (63%) were DCs. Panelists included 1 MD anesthesiologist, 2 MDs in other medical specialties, 2 RNs who work on MUA teams, 6 DCs who practice MUA, 4 DCs who do not practice MUA, and 1 attorney. Of the DCs, all were practitioners and 5 were also on the faculty of 5 different chiropractic colleges. There were 13 (81%) male and 3 (19%) female panelists, with a mean of 23 years professional experience (median 25 years). States represented were CA (5), FL (4), TX (2) and 1 each from GA, NC, NY and RN; one panelist resides in Malaysia. Most of the DCs were broad-scope in terms of practice approach, meaning that they utilized a number of procedures in addition to manipulation [21].

### Delphi process

The Delphi process was conducted by e-mail. Each set of seed statements to be rated was identified by an ID number. Only the project coordinator could link the ID to the panelist’s names, for purposes of distribution and follow-up. The Delphi process was conducted in a blinded manner, so that neither the panelists nor the core committee knew the identity of the raters or those who had made any individual comments, during the development of consensus. We used the widely-used and well-established RAND-UCLA consensus process methodology in rating the seed statements [22]. We used an ordinal rating scale ranging from 1 (highly inappropriate) to 9 (highly appropriate). We explained that by “appropriateness” (as specified by RAND/UCLA) [22], “we mean that the expected health benefit to the patient exceeds the expected negative consequences by a sufficiently wide margin that it is worth doing, exclusive of cost” [22].

In scoring, ratings of 1-3 indicated “inappropriate”; 4-6 “undecided”; and 7-9 “appropriate”. Panelists rating a statement as “inappropriate” were required to give a specific reason and, if possible, provide a reference from the peer-reviewed literature to support it. There was unlimited space provided for panelists to make comments, and the project coordinator entered all comments into a Word file, identified by ID number, rating and seed statement number. The project coordinator entered the numerical ratings into an SPSS v. 21.0 database and one of the investigators (CH) analyzed the results, computing the median rating and percentages of agreement for each statement. We considered consensus present when both the median rating was 7 or higher and at least 80% of the panelists gave a rating of 7 or higher. Rounds were to be repeated until consensus was reached.

The core committee reviewed all comments and revised the statements on which consensus was not reached, based on the panelists’ comments. The project coordinator then circulated the revised statements, along with the de-identified comments, to the entire panel for the next round.

### Delphi process and panelist summary

The Delphi process was conducted from August-October 2013. Fifteen panelists of the 16 participated in each of the two Delphi rounds, although one panelist of the 16 participated in only Round 1 and a different panelist only participated in Round 2. Consensus on all was reached on 38 of the 43 statements after one round, and consensus on the remaining 5 statements after the second round.

### Results: consensus guidelines

The following section contains the consensus-based guideline statements. It provides the statements that are the result of the consensus process, which is therefore the complete guideline on for the practice and performance of manipulation under anesthesia.

### General guideline disclaimer

This guideline is intended for practitioners, facilities, and other interested parties. Decisions to adopt particular courses of action must be made by trained practitioners on the basis of the available resources and the particular circumstances of the individual patient. This guideline is not to be applied to any specific patient, in any manner, and any decision requiring necessary testing, patient candidacy or follow-up procedures must be made by the individual doctor and determined by the needs of the patient. Safety and effectiveness should drive the doctor’s decision when considering Manipulation Under Anesthesia protocols. This guideline is not intended for utilization review purposes. The American Association of Manipulation Under Anesthesia Physicians denies responsibility for any injury or damage resulting from actions taken by practitioners after considering this guideline.

### Protocols and standards

#### Patient selection: *clinical candidacy for MUA*

The following factors qualify a patient for clinical candidacy for MUA.

• The patient has undergone an adequate trial of appropriate care, usually including spinal manipulation by a chiropractor, and often with medical co-management, and continues to experience intractable pain, interference to activities of daily living, and/or biomechanical dysfunction.

• Sufficient care has been rendered prior to recommending MUA. A sufficient time period is usually considered a minimum of 4-8 weeks, but exceptions may apply depending on the patient’s individual needs. Most patients selected for MUA procedures have had longer courses of care, but those with more severe symptoms and little or no response to conservative management are best considered sooner than later to avoid unnecessary additional costs and increased suffering.

• Physical medicine procedures have been utilized in a clinical setting during the 6-8 week period prior to recommending MUA.

• The patient’s level of reproduced pain interferes with activities of daily living or causes disability (that is, the inability to fully participate in work and other activities).

• Diagnosed conditions must fall within the recognized categories of conditions responsive to MUA. The following disorders are classified as acceptable conditions for utilization of MUA:

1) Patients for whom manipulation of the spine or other articulations is the treatment of choice; however, the patient’s pain threshold inhibits the effectiveness of conservative manipulation.

2) Patients for whom manipulation of the spine or other articulations is the treatment of choice; however, due to the extent of the injury mechanism, conservative manipulation has been minimally effective during a minimum of 4-8 weeks of care and a greater degree of movement of the affected joint(s) is needed to obtain patient progress.

3) Patients for whom manipulation of the spine or other articulations is the treatment of choice by the doctor; however, due to the chronicity of the problem and/or the fibrous tissue adhesions present, in-office manipulation has been incomplete and the plateau in the patient’s improvement is unsatisfactory.

4) When the patient is considered for surgical intervention, MUA is an alternative and/or an interim treatment and may be used as a therapeutic and/or diagnostic tool in the overall consideration of the patient’s condition.

5) When there are no better treatment options available for the patient in the opinions of the treating doctor and patient and in consideration of the cause of the patient’s related pain, impairment, and/or disability.

### Diagnosis

#### Establishing medical necessity

Every condition treated must be diagnosed and justified by clinical documentation in order to establish medical necessity. Documentation of the patient’s progress and the patient’s response to treatment are combined to confirm the working diagnosis. Those diagnoses which are most responsive to MUA include, but are not limited to the following:

• Sclerotogenous pain from the medial branch of the dorsal rami.

• Cervical, thoracic, lumbar, sacroiliac, and sacrococcygeal sprain/strain subluxations (neuromechanical dysfunctions) with or without resultant myofascial pain syndromes.

• Intervertebral disc syndromes without fragment, sequestration, or any contraindication to in-office manipulative procedures and with or without radiculopathy.

• Cervical brachial pain syndrome.

• Chronic recurrent cervicogenic headaches, after ruling out pathologic etiologies (for example, organic brain syndromes, or other vascular or neurological syndromes).

• Failed back surgeries with adhesion formation in a patient who has not adequately responded to clinical therapeutic trials of manipulation, traction, and soft tissue techniques (including but not limited to myofascial release).

• Adhesive capsulitis and/or soft tissue contractures relative to articular motion of the appendicular skeleton, e.g. shoulder and knee.

• Functional biomechanical dysfunction syndromes (including but not limited to sprain/strain with fixation and vertebral subluxation complex). Functional radiography and particularly lateral bending, weightbearing radiographs are recommended, as clinically indicated, to detect and characterize intersegmental motion restrictions in the spine.

### Frequency and follow-up procedures

#### Determining the necessity and frequency of MUA

The following should be considered when determining the necessity and frequency of manipulation under anesthesia:

• Patient’s response and progress to previous conservative care.

• Consideration of activities of daily living and disability.

• Patient’s psychological acceptance of the MUA procedure, and psychosomatic response to overcoming chronic pain and discomfort.

• Prevention of additional gross deterioration.

• Prevention of possible surgical intervention.

• Chronicity.

• Length of current treatment and patient progress.

• Patient’s age.

• Number of previous injuries to the same area.

• Level of pain considering standard 4-8 week minimum protocol parameters and deciding whether a variation from the guidelines may be appropriate for the individual patient’s needs.

• Patient’s tolerance of previous treatment procedures and their success or failures.

• Muscle contraction level (beyond splinting).

• Response to previous MUA’s based on objective clinical documentation and protocols for determining patient progress.

• Fibrous adhesion from failed back surgery or prior injury.

• Patient willingness and availability to participate in appropriate post-procedures follow-up to optimize results.

#### Protocols for determining the frequency of the MUA procedure

A treatment plan of three consecutive days of treatment is recommended, on the rationale that serial procedures allow a gentler yet effective treatment plan with better control of biomechanical force resulting in increased safety, and more focused and effective subsequent procedures after monitoring the effects of those administered previously.

Ranges of motion should always be measured after an appropriate warm-up period for consistency and as recommended within the American Medical Association Impairment Guidelines.

• Single spinal MUA is most often recommended for younger patients; when the area to be treated has not been previously injured; and when the verifiable global and intersegmental motion restrictions are relatively mild.

• Single spinal MUA is most often recommended when conservative care has been rendered for a sufficient time (usually a 4-6 week minimum) and the patient’s activities of daily living or work activities are interrupted in such a fashion as to warrant a more aggressive approach.

• If the patient is treated for intractable pain with a single MUA procedure and responds with 80% symptomatic and functional resolution, the necessity for future MUA’s should be considered and depends in part on the objective parameters determined during and after the MUA procedures.

• Serial MUA is recommended when the patient’s condition is chronic and when conservative care as described in this guideline has been rendered.

• Serial MUA is recommended when the injury is recurrent in nature and fibrotic tissue and articular fixation prevents a single MUA from being optimally effective.

### Parameters for determining MUA progress

Parameters for determining MUA progress may include, but are not limited to:

• Subjective changes

• Patient’s pain index, visual analogue scale, faces of pain.

• Patient’s ability to engage in active range of motion.

• Patient’s change in activities of daily living.

• Patient’s change in job performance.

• Objective changes

• Change in measurable muscle mass, function, and strength.

• Change in muscle contractibility.

• Change in EMG and/or nerve conduction studies.

• Change in controlled measurable passive range of motion.

• Change in diagnostic studies (X-rays, CT, MRI), including functional radiography.

### General post MUA therapy

#### Therapy following first MUA

• Repeat MUA stretching.

• Physiotherapeutic modalities as indicated by patient presentation.

• Patient to rest at home with walking and range of motion exercises encouraged to patient tolerance.

#### Therapy following subsequent MUAs

• Same as 1st day.

• No further manipulation should be required.

• May add proprioceptive neurofacilitation protocols. These can be incorporated during stretching if tolerated.

#### Therapy following last MUA

• Same protocol as above with proprioceptive neurofacilitation.

• Additional home instructions to include range of motion and strengthening exercises as condition permits and to patient tolerance can be provided to the patient at this time.

#### Follow-up therapy following MUA—one week after last MUA

Treatment frequency during the first week should be 3-4 days dependent on the individual patient’s needs. These follow-up procedures should include all fibrosis release and manipulative procedures performed during the MUA procedure to help prevent re-adhesion.

#### Follow-up therapy following MUA—weeks 2 and 3 after last MUA

• Continue full protocols to include fibrosis release procedures, proprioceptive neurofacilitation, and manipulative procedures as needed to maintain global and intersegmental motion improvements obtained during the MUA procedure.

• Begin home rehabilitation exercises 2-3 times per week.

#### Follow-up therapy following MUA—weeks 7-8 after last MUA

• Continue full protocol (fibrosis release procedures, proprioceptive neurofacilitation and manipulative procedures).

• Patient treated 1-2 times per week for 4-5 weeks depending on patient needs.

• Active progressive resistive strength/stabilization exercises, supervised/unsupervised 2-3 times per week; optimal rehabilitative procedures should include attention to aerobic, flexibility, strength, and coordination considerations.

### Safety

Physicians and co-attending doctors should be appropriately certified. Both patient and doctor safety are important factors to be taken into consideration.

#### Patient safety

MUA is performed using the anesthesia techniques determined by the anesthesiologist to be appropriate for the patient. MUA is performed with the patient in a sedated state as determined safe and effective by the attending anesthesiologist. The chiropractic providers do not make any decisions regarding the medical management nor do they direct or use any of the medications required by the anesthesiologist during his or her medical management.

The primary doctor and the co-attending doctor move the patient into specific ranges of motion to accomplish the procedure. In this capacity, the patient depends on the primary doctor and co-attending doctor to protect them from bodily injury. Since the patient is only minimally responsive to painful stimuli and does not have the ability to respond to immediate proprioceptive input, both the primary doctor and the co-attending doctor are key to a safe and successful procedure.

The co-attending doctor is responsible for patient stability, patient movement, patient observation, and completing portions of the procedure should the primary doctor need assistance or become unable to perform the procedure. Since there are several instances during the procedure when the primary doctor has to move the patient, stabilizing and working with the patient would be unsafe without assistance from another doctor competent and knowledgeable in MUA.

#### Doctor safety

Manipulation under anesthesia is a very physically demanding therapeutic procedure. Since the patient is in a sedated state, the doctor has the added responsibility of insuring that the patient’s extremities and torso do not fall from the treating surface. The doctor must also be able to move the patient without the assistance of the patient.

The co-attending doctor is an integral part of this procedure and is responsible for helping the primary doctor move the patient through the prescribed ranges of motion. The co-attending doctor is present to insure that all movements are accomplished without injury to the patient or to the primary doctor performing the procedure. As a result of the added potential risk to the patient in a sedated state, there is a high risk of injury to the doctor and the patient if only one doctor were to attempt the complex techniques necessary for the MUA procedure. Inclusion of a co-attending doctor, who is a certified MUA practitioner, is the safest way to perform this procedure. It may be unsafe to perform an MUA without a competent and knowledgeable MUA doctor as the co-attending doctor and anything other than allowing another MUA certified doctor to act as a co-attending doctor imposes potential risks. By using a certified MUA practitioner as a co-attending doctor, optimal effectiveness and safety standards are maintained. This is proper standard of care policy for the MUA procedure and needs to be recognized as such by anyone recommending MUA, or reimbursing for MUA.

In the cervical spine, the co-attending doctor must secure the patient’s shoulders and provide counterforce procedures to obtain the necessary traction for this part of the procedure. In the thoracic spine, the co-attending doctor turns the patient, stabilizes the patient and applies proper counter traction for the MUA maneuvers. In the lumbosacral area, the co-attending doctor coordinates movements with the primary doctor, assists with the actual procedures, and can complete the MUA procedures as necessary. Procedure efficacy is enhanced when both doctors are trained and knowledgeable regarding the appropriate forces and counterforces required to perform safe and effective MUA procedures.

A certified MUA physician carries the appropriate malpractice insurance to perform MUA and so does his or her co-attending doctor. Since non-certified assistants may not carry malpractice insurance for MUA, utilization of ancillary staff to assist with the MUA procedure may potentially place the entire team and the facility at risk. Therefore, only a certified MUA practitioner should co-attend the MUA procedure.

#### Facilities

All MUA procedures should be performed in the highest quality facility available and within the parameters of state regulations. MUA should only be performed in hospitals, ambulatory surgery centers or other specialty centers that meet the American Society of Anesthesiology standards, and adhere to recognized standards of care.

### Compensation

Fees must be reasonable and in relation to standards and relative values within each state. The CPT codes used for spinal MUA include but are not limited to 22505, 20999, 23700, 27275. It is recommended that chiropractic/medical necessity and authorization be obtained prior to scheduling the patient. Fees should be reasonable and in keeping with standard fee structures.

### Anesthesia standards for outpatient MUA

• Anesthesia is provided under the direct supervision of a board-certified anesthesiologist or other osteopathic or medical physician based on applicable state law. The MUA certified chiropractors limit their involvement to procedures within their scope of practice which may vary from state to state.

• The anesthesia provided must adhere to guidelines and recommendations accepted in his/her community for delivering anesthesia to patients.

#### Pre-MUA anesthetics procedures

• Patients are appropriately evaluated by their chiropractic or MUA doctors to assess candidacy prior to the procedure. Anesthesiologists will typically perform a history and physical prior to the procedure and may elect to not go forward with and may cancel the procedure if they feel that the patient might be at risk from a medical standpoint.

• All appropriate clearance forms, laboratory results, imaging reports and other supported data are available for review in the patient’s chart. Special testing should be provided only as deemed necessary and based on individual needs. Since the fibrosis release from manipulative procedures performed during MUA carries similar risks as chiropractic in-office procedures, the need for diagnostic tests is commonly determined using similar criteria as might be performed during in-office care with physical methods. Individual laboratory testing or special testing requirements may differ from state to state or from facility to facility.

#### Intra-MUA anesthetics procedures

• The anesthesiologist selects the anesthesia based on the patient’s medical condition and is responsible for all medical decisions.

• The chiropractic doctor does not order or administer any medications.

• Blood pressure, oxygen saturation and EKG are recorded by the anesthesiologist, or at his/her direction, throughout the procedure.

• Supplemental oxygen is available in case it is needed.

• Resuscitate equipment and medications must be readily available at all times.

• An emergency facility must be available locally pursuant to state and accreditation agency requirements.

#### Post-MUA anesthetics procedures

• The anesthesia provider is responsible for the medical discharge of the patient.

• Once the patient is stable, the anesthesia provider may depart as long as there is a trained Advanced Cardiac Life Support (ACLS) provider present in the facility and pursuant to local regulations and patient needs.

### Nursing standards—patient care responsibilities

#### Pre-MUA nursing patient care responsibilities

• Witness signature of procedure consent.

• Verify and document NPO compliance.

• Verify responsible adult driver or escort is available for the patient.

• Verify and document present medications and allergies.

• Direct and assist the patient with appropriate attire for procedure.

• Escort the patient and medical chart to procedure room.

#### Intra-MUA nursing patient care responsibilities

• Direct and assist patient in transferring to the procedure table.

• Maintain patient safety, privacy and dignity.

• Complete appropriate medical record forms.

• Be available to assist anesthesia provider as needed.

• Be available to assist MUA providers as needed.

• Assist in transferring the patient to a recovery bed.

• Raise the bed’s side rails for patient safety as required.

#### Post-MUA nursing patient care responsibilities

• Transport patient to recovery room with anesthesia provider.

• Receive report from anesthesia provider including medications given, vital signs, IV history and any other pertinent information.

• Secure appropriate monitoring equipment.

• Record vital signs on admission to recovery area and every 15 minutes until stable and then every 30 minutes until discharge.

• When the patient is conscious and alert, oral fluids may be offered.

• When the patient is tolerating fluids, a light snack may be offered.

• When the patient is tolerating foods and fluids well and vital signs have remained stable for 15 minutes, the IV/heparin lock may be discontinued.

• The patient may then be discharged to their responsible adult escort/driver with written instructions for activity and follow-up care.

## Discussion

Similar to many other treatments available for spinal conditions, MUA does not have the unequivocal support for effectiveness and efficacy that would be provided by multiple randomized controlled trials and meta-analyses. If proven alternatives that addressed these same conditions were available, other choices would be recommended prior to considering MUA.

However, there is a fair amount of lower-level evidence in regards to the safety and efficacy of this procedure. This led Dagenais et al. in their systematic review to state: “However, almost all studies to date on these procedures have reported positive results, indicating that patients who undergo their procedures have a reasonable prognosis” [3], ^p. 148^.

In the absence of strong evidence, this guideline was designed to provide recommendations on best practices of MUA for interested and affected parties; namely, patients, doctors, and payers.

When a doctor or patient considers MUA, he/she is commonly comparing the appropriateness of this procedure to many other procedures with a similar evidence level for support. Kohlbeck, et al., in their systematic review expressed this consideration for practitioners:

“Medicine-assisted spinal manipulation therapies have a relatively long history of clinical use and have been reported in the literature for over 70 years. However, evidence for effectiveness of these protocols remains largely anecdotal, based on case series mimicking many other surgical and conservative approaches for the treatment of chronic pain syndromes of musculoskeletal origin. There is, however, sufficient theoretical basis and positive results from the case series to warrant further controlled trials on these techniques” [2], ^p. 288^.

Payers are also faced with challenges when considering reimbursement for MUA procedures. This is also summarized by Kohlbeck, et al. as follows:

“If a clinician recommends or offers, and a payer reimburses, surgery, injections, epidurals, and certain physical therapy approaches, to a patient without requiring substantial proof of effectiveness and safety, then it would be difficult to deny the use of medication-assisted manipulation or fail to reimburse for it… It would seem unreasonable, however, to hold medication-assisted manipulation to a higher standard of scientific rigor than that required of other treatment approaches” [2], ^p. 301^.

The Delphi panel who developed this guideline was composed of experienced physicians, nurses and educators, both practitioners of MUA and practitioners who do not practice MUA but are experienced in the treatment of spine-related pain. This group reached a high level (80%) of consensus on recommendations related to the practice of MUA. This lends clinical validity to the recommendations and therefore should guide MUA practitioners. This guideline is not intended to be prescriptive, or to suggest that MUA is the only therapy of choice when seeking relief for spinal dysfunction and pain. It is intended to provide practitioners with evidence-informed, consensus-based parameters guiding the use of MUA.

## Competing interests

The American Association of Manipulation Under Anesthesia Providers provided consultant fees for Dr. Hawk’s role on the project. She served as an independent contractor to the project, which is not associated with her position at Logan University. RG and EC have no financial interest in any part of the process and have not received any remuneration for their part in this project. Both RG and EC practice manipulation under anesthesia and teach it in post-graduate education. The authors declare that they have no competing interests.

## Authors’ contributions

RG developed the original seed statements, contributed to the literature search, participated in conducting the Delphi rounds, and was the primary author of the paper. EC also developed the original seed statements, contributed to the literature search, participated in conducting the Delphi rounds, and contributed to writing the paper. CH conducted the Delphi rounds, contributed to the literature search and contributed to writing the paper. All authors read and approved the final manuscript.
